# Experimental Modeling, Statistical Analysis, and Optimization of the Laser-Cutting Process of Hardox 400 Steel

**DOI:** 10.3390/ma17122798

**Published:** 2024-06-07

**Authors:** Mehdi Safari, Seyed Mohammad Abtahi, Jalal Joudaki

**Affiliations:** Department of Mechanical Engineering, Arak University of Technology, Arak 38181-46763, Iran

**Keywords:** laser-cutting process, Hardox 400 steel, surface roughness, kerf width, design of experiments

## Abstract

Fiber laser cutting machines are widely used in industry for cutting various sheet metals. Hardox steel is widely used in the construction of machinery and equipment that are subjected to wear and impact due to its anti-wear properties and good impact resistance. In this experimental study, the effect of input parameters including laser output power (LOP), laser-cutting speed (LCS), and focal point position (FPP) of fiber laser on the surface roughness and kerf width of Hardox 400 steel sheets are studied. In addition, the optimization of input parameters to achieve the desired surface roughness and kerf width are investigated and analyzed using the response surface methodology (RSM). The experiments are performed using a 4 kW fiber laser-cutting machine and the output results including surface roughness and kerf width are measured using roughness meters and optical microscope. The results of the analysis of variance (ANOVA) for surface roughness and kerf width show that the FPP and LCS are the most significant process parameters affecting the surface roughness and kerf width. With a positive focal point, the surface roughness decreases while the kerf width increases. With increasing the laser-cutting speed, both the surface roughness and kerf width decrease.

## 1. Introduction

The laser-cutting process (LCP) for sheet metals has increased significantly in recent years due to its high production speed and cutting surface accuracy, which leads to improved industrial production processes. The LCP is a non-contact method that utilizes the energy of a focused laser beam without the use of hard tooling, making it easy to cut complex geometries. With this method, the challenges of cutting hard materials using conventional methods are eliminated, the heat-affected zone (HAZ) is very small, the kerf width is very small, and there is very little residual stress and distortion [1,2]. Most researchers prefer to consider the main parameters affecting the LCP in their research, which include the intensity of the beam power, the diameter of the laser beam, the type and pressure of the assistant gas, the pulse frequency, the cutting speed, and the position of the focal point relative to the workpiece surface. This is because these parameters can be easily controlled and are the most effective parameters for measuring cut quality. The most common output parameters that indicate surface quality and have been studied by researchers are surface roughness and kerf width [2]. Hardox steel is a trademark that encompasses a wide range of high-strength, wear-resistant steels that retain their physical and mechanical properties over a wide temperature range and are widely used in industrial applications. These steels are used in environments involving severe wear and impact due to their exceptional toughness, hardness, and high wear resistance [3,4]. Hardox 400 steel has high wear resistance, fatigue resistance, and impact resistance. Due to these properties, it is widely used in earthmoving and mining equipment, agriculture, recycling, cement, and concrete production. For example, it is a reliable choice for the production of dump truck bodies, stone crusher equipment, bulldozer blades and concrete mixers, and agricultural and waste equipment due to the harsh working conditions [5]. Due to the physical and chemical properties of this steel, the use of traditional machining leads to rapid tool wear with an average surface roughness [6,7,8]. Since this steel exhibits high thermal conductivity, melt viscosity, and absorption then the LCP can be an efficient and suitable alternative process [9,10]. In recent years, researchers have investigated and studied the possibility of LCP of various materials. Very few researchers have investigated and studied laser-cutting conditions on Hardox steel. Gheorghe and Girdu [11] conducted an experimental study on CO_2_ laser cutting of Hardox 400 steel and the effect of process parameters including laser output power (LOP), gas pressure (GP), and laser-cutting speed (LCS) on the kerf width based on a full factorial design to improve kerf characterizations. They found that LOP has the most significant effect on kerf width. In the interaction between LOP and GP at constant LCS, the minimum kerf was obtained when setting the LOP and GP to intermediate values. In another study by the same researchers [12], they optimized LCP parameters to increase process productivity and reduce energy costs on Hardox 400 steel. The findings of this study show that cutting efficiency is highly influenced by LCS and subsequently by LOP, emphasizing the importance of optimizing LCS and LOP combinations to achieve cost-effective and efficient production processes. Mileșan et al. [13] conducted an experimental study on the LCP of Hardox 400 steel sheets and showed that low surface roughness is obtained at high speeds and low powers, and increasing the LOP leads to an increase in laser focus energy density and surface roughness. Gondalia and Sharma [14] investigated the effect of various LCP parameters such as LCS, LOP, GP, and pulse frequency on Hardox 400 with a thickness of 8 mm using oxygen gas. This study was conducted to establish relationships between these parameters and improve cut quality, which includes surface roughness and kerf. Prajapati et al. [15] investigated the effect of CO_2_ laser machine parameters such as LOP, GP, LCS, and thickness on the surface roughness of Mild Steel and Hardox 400. The experiments were designed based on the Taguchi L27 orthogonal array with three different levels of each input parameter. ANOVA was performed to interpret the results. The results showed that LCS and workpiece thickness plays a significant role in surface roughness. Design of experiments (DOE) using the response surface method (RSM) is a mathematical and statistical method used to model and analyze problems in which one response (output variable) is affected by several independent variables (input variables). The goal of RSM is to optimize the response by finding the best combination of input variables, which involves using designed experiments to collect data, fitting models to the data, and using optimization techniques to find the combination of variables that maximizes or minimizes the response. This method helps to understand the effect of LCP on cut quality and predict the results of different parameter settings by developing regression models and using statistical analysis such as ANOVA. It is also very efficient for optimizing LCP, as it can handle complex and nonlinear relationships and interactions between parameters that are common in LCP. It also allows for the optimization of multiple responses simultaneously, which is important for achieving a balance between different aspects of cut quality [16,17,18]. Nguyen et al. [18] in an experimental and comparative study to optimize LCP parameters on Stainless Steel 304 concluded that the RSM predicts optimal conditions more accurately than Taguchi. The RSM was strongly recommended for identifying optimal parameter settings and interactions, and the Taguchi method can be a suitable method for screening important variables for cases where experimentation is costly and time consuming. Sharma and Kumar [19] successfully applied the RSM using the Box–Behnken design (BBD) with analysis of LCP parameters on the responses of laser cutting of aluminum metal matrix composite and optimized it, validating the predicted model with experimental data and showing the importance and accuracy of the model. Wang et al. [20] discuss LCP for nickel-based superalloys in an experimental study and investigates the LCP parameters and their effects on surface roughness using the RSM. The results of the ANOVA showed that the data fit well with the predicted nonlinear regression model. The main parameters of LCS, LOP, and focal length had the most significant effect on the temperature around the cutting area, in that order. Vishnu Vardhan et al. [21] investigated the experimental process parameters of LCP including LOP, LCS, and GP to improve the surface quality of SS 314 stainless steel. The LCP parameters were optimized using the RSM to minimize surface roughness. Eltawahni et al. [22] investigated the LCP of AISI316L stainless steel by process parameters such as LOP, LCS, FPP, nitrogen pressure, and nozzle diameter and applied the RSM to develop the mathematical models and optimize the kerf width, surface roughness, and operational cost. Vora et al. [23] studied the advantages of fiber laser for the LCP of titanium alloy Ti6Al4V. The LOP, LCS, and GP were selected as the input parameters, and surface roughness, kerf, bead height, and material removal rate (MRR) were selected as the output variables. The effects of the input variables were analyzed through ANOVA, main effect plots, residual plots, and contour plots. Safari et al. [24] conducted an experimental study on the LCP of polymethyl methacrylate (PMMA) using the RSM to analyze the effects of process variables on surface roughness, kerf, and taper angle. The results showed that LBD has a significant effect on surface roughness, while LOP and LCS affect kerf and taper angle. Multi-objective optimization was also used to determine the optimal conditions and minimize surface roughness, kerf, and taper angle. Jadhav and Kumar [25] investigated the effect of process parameters including LOP, LCS, and GP on surface roughness using RSM in an experimental study on LCP of AISI 304 stainless steel with an optimization process to minimize surface roughness. Kotadiya et al. [26] investigated the LCP of stainless steel in an experimental study and analyzed process parameters including LOP, LCS, and GP using RSM to optimize responses including surface roughness and kerf, and it was found that LOP has the most significant effect on the responses.

To the authors’ knowledge, few pieces of research have been reported on the LCP of Hardox 400 steel, especially with a fiber laser source. The Hardox 400 steel is widely used in various industries due to its special physical and chemical properties. It should be noted that laser cutting has been used in industries for more than 30 years but still, it has some unknown aspects that lead to unoptimized cutting. The irradiated heat produces microstructural changes and a little deviation in flatness (usually bowing and wrapping) but the manufacturers cut the sheets with these unwanted phenomena because of its speed and low cost (good productivity). By the way, laser cutting is used widely without thinking about these defects. This manuscript aims to find the cutting condition that minimized the surface roughness and minimized kerf width. This article is an attempt to focus on surface roughness and kerf width in laser cutting. Therefore, in this work the LCP of Hardox 400 steel will be studied and the effects of some important process parameters such as laser output power (LOP), laser-cutting speed (LCS), and focal point position (FPP) of the surface roughness and kerf width are investigated.

## 2. Materials and Methods

**Figure 1:**
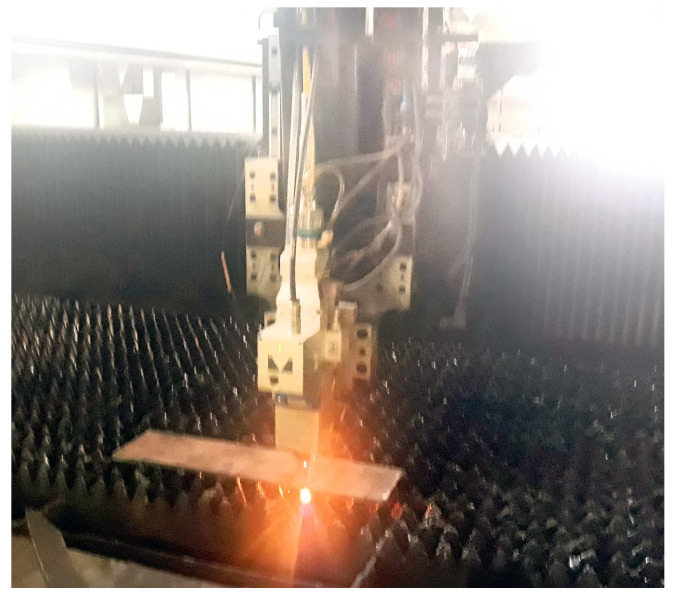
Fiber Laser Machine for LCP of Hardox 400 steel.

The experiments were carried out with an industrial fiber laser machine with a maximum power of 4 kW (Figure 1). The examined material is a Hardox 400 steel sheet (SSAB Steel industry company, Stockholm, Sweden) with a thickness of 8 mm. For implementing the tests, a 330 mm × 130 mm sample was prepared. The steel was provided by a certified steel supplier and the chemical composition of the Hardox 400 steel sheet is shown in Table 1.

In this study, the RSM (Box–Behnken type) was used to investigate the relationship between input variables and output responses. To investigate the changes in surface roughness and kerf width, the input variables of laser output power (LOP), laser-cutting speed (LCS), and focal point position (FPP) were selected as influential factors of the LCP based on a review of previous research [12,14,15,24]. Accordingly, the experimental design was performed using three parameters and three different levels, as shown in Table 2. The effect of process parameters and their interactions were analyzed using analysis of variance (ANOVA), the mathematical model was determined based on the results of the experimental design, and finally, process variables were optimized. Minitab software (Minitab 18.1) was used for experimental design and data analysis, and the list of experiments according to RSM is shown in Table 3. It should be noted that different DOE techniques exist, such as full-factorial and Taguchi. The full-factorial design is used when the effect of input parameters is an always ascending or always descending function and it is usually used as a 2-level full-factorial DOE design. So, the data will be analyzed by 2^3^ = 8 experiments. When the researcher guesses that the output variable firstly increases and then decreases (or vice versa) by the input parameter, it is necessary to use the RSM method. Also, the effect of the interaction of the input variables and the square of the input variables can be discussed in this method. Using a 3-level full-factorial DOE design can lead to including higher-order terms such as cubic terms and their third-order interactions and some fourth-order terms in the calculation, which is very complicated and is avoided by the researchers. So, the RSM is used for DOE in this study.

**Figure 2:**
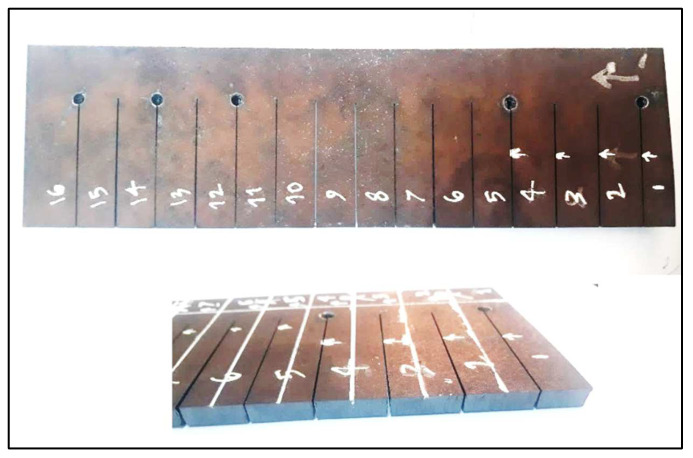
The laser cut specimens after LCP.

In Figure 2, the straight cuts with a length of 80 mm were made on a Hardox 400 steel sheet according to the list of experiments designed based on RSM (Table 3).

**Figure 3:**
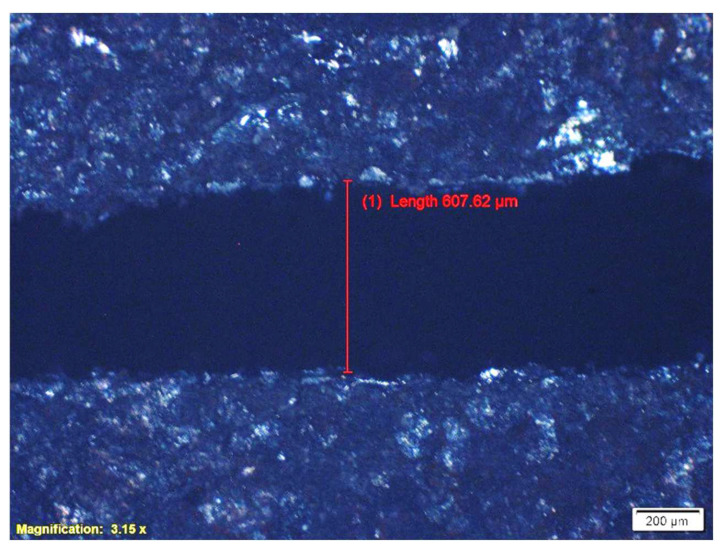
Measurement of kerf width using OM software (DP73 Firmware (Ver. 2.116)).

OLYMPUS DP73 optical microscope (OLYMPUS, Tokyo, Japan) with 100× magnification was used to measure the kerf width. In Figure 3, a sample of kerf width measurements using the optical microscope is shown. The kerf width was measured at five points and the average of the data was used for data analysis using Minitab software.

**Figure 4:**
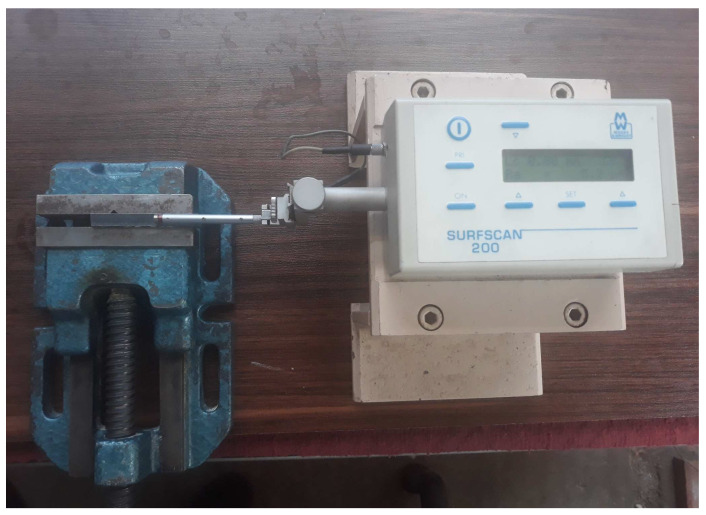
The surface roughness measurement apparatus.

A surface roughness instrument (Surfscan200) (Surfscan, Rocklin, CA, USA) was used to measure the surface roughness (Figure 4). The nominal accuracy of this device is 0.001 µm, and the stylus probe scans the surface with 4 mm as evaluation length (0.8 mm sampling length or cut-off length × 5 sampling lengths) and 0.4 mm pre-travel and 0.4 mm post-travel lengths. The average surface roughness value (Ra) is calculated according to the ISO 21920-3:2021 standard [27]. To measure the surface roughness of the samples, the measurements were carried out in three zones of the surface for each sample. The instrument probe scans the surface roughness along the direction of the laser-scanning direction, and the average value was considered as the final surface roughness of the cutting groove.

## 3. Results and Discussion

To determine the effect of each parameter and the interactions of the parameters on the surface roughness and kerf width, ANOVA was used. It should be noted that in the ANOVA results, a *p*-value less than 0.05 shows that the investigated parameter is effective on the expected output. In addition, in the ANOVA results, the R-sq and R-sq (adj) values are also provided, which indicate that the data fit the regression model and are of very high accuracy.

### 3.1. Surface Roughness Analysis

**Figure 5:**
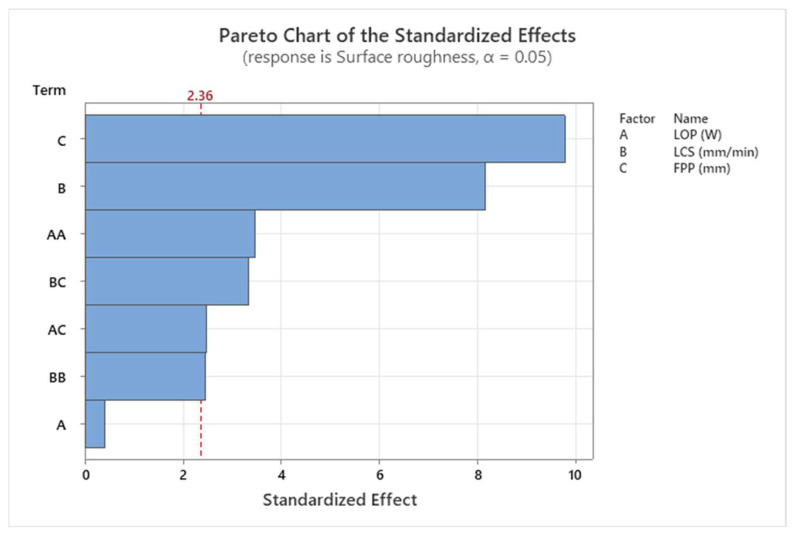
Pareto chart for surface roughness.

The results of the ANOVA for surface roughness are shown in Table 4. FPP and LCS are the effective parameters in the process, while LOP has the least effect. The results of the ANOVA for surface roughness are also shown graphically in a Pareto chart in Figure 5. The red line in Figure 5 is called the reference line and the variable that crosses the line is a significant parameter. It is calculated from the T-distribution function.

**Figure 6:**
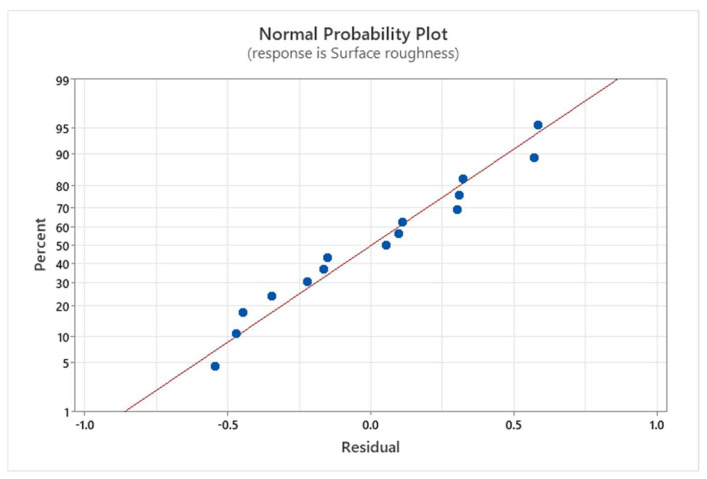
Normal Probability Plot for the surface roughness.

Figure 6 presents the Normal Probability Plot for the surface roughness. Observing that the data points align approximately linearly along the regression line, we can conclude that the data follow a normal distribution and that the linear regression model fits the experimental data well. The distance of points from the red line (Residual) shows the quality of interpolation, and the coefficient of determination (R^2^) is calculated according to the closeness of the points to the line. From Table 4 it can be reported that the R^2^ is 96.56% for surface roughness.

Based on the obtained results, the regression equation for the prediction of surface roughness for LCP of Hardox 400 steel sheet is given by Equation (1):

Surface roughness = 49.6 − 0.02571 LOP − 0.02146 LCS + 5.72 FPP + 0.000006 LOP × LOP + 0.000007 LCS × LCS − 0.001625 LOP × FPP − 0.002917 LCS × FPP(1)

**Figure 7:**
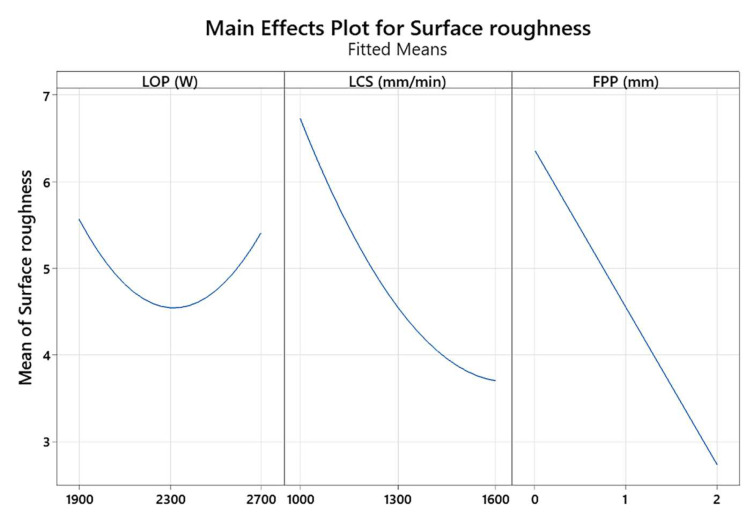
Main effect plot for surface roughness.

The effect of the main parameters such as LOP, LCS, and FPP on the surface roughness is shown in Figure 7. In this research, the focus position has been set between 0 and 2 mm above the surface of the sheet. As is seen in Figure 7, with the increase in laser power, the surface roughness decreases due to the entry of more thermal energy into the cutting area and complete melting in this area. Consequently, with a further increase in laser power, due to the destruction of more areas of the sheet in the cutting area, the roughness of the cutting surface increases. In addition, with increasing the LCS, surface roughness decreased. This is because the thermal energy has less time to melt the cut surface and increase surface roughness. The laser energy density should also be applied to the workpiece surface in an appropriate range and for a specified time to prevent an increase in surface roughness.

**Figure 8:**
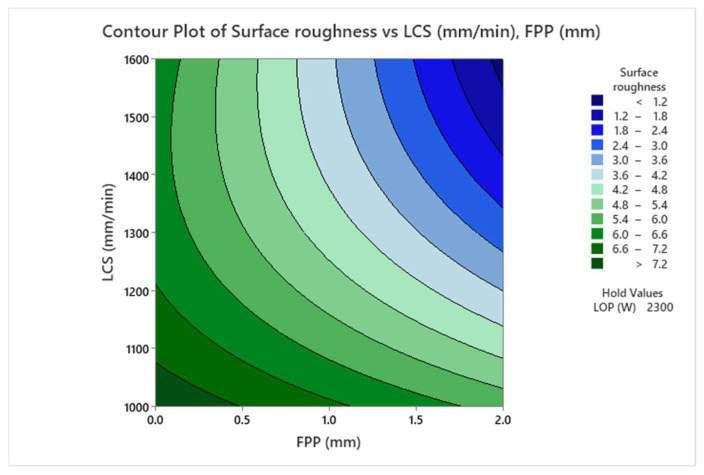
Contour Plot of the Interaction Effects of FPP and LCS on Surface Roughness.

It is concluded from Figure 7 that the surface roughness is decreased with increasing the distance from the focal point of the laser beam from above the surface of the sheet. The reason is that increasing the positive focus allows the gas to react better with the melt in the front of the cut, generating additional heat in the cut gap, melting it, and moving it down the gap, resulting in a smoother surface. Figure 8 shows the contour plot of the interaction effects of FPP and LCS on the surface roughness of the cutting area of the sheet. It is found from Figure 8 that with increasing positive FPP and LCS, the surface roughness decreases. As the FPP increases, the focus of the laser’s heat beam is positioned above the workpiece, allowing the oxygen gas to react thermally with the workpiece, adding a thermal energy source to the cutting process that melts the material. With increasing speed, the molten material is expelled from the cutting groove along with the oxygen GP, improving the surface quality of the cut.

### 3.2. Kerf Width Analysis

**Figure 9:**
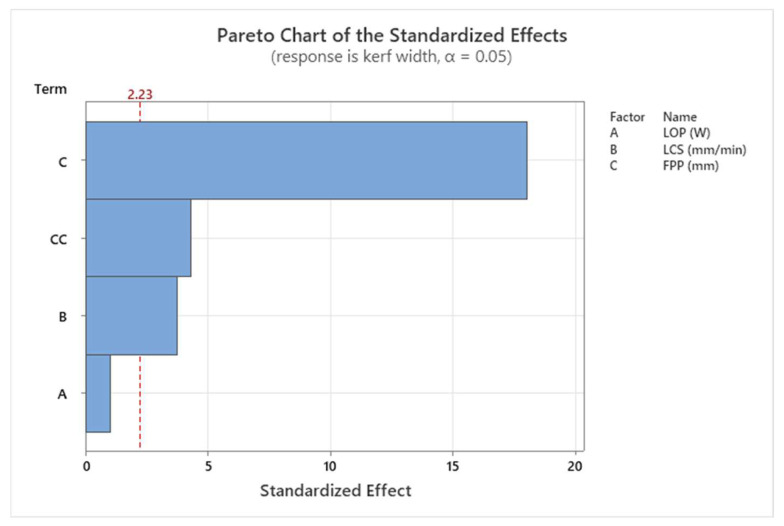
Pareto chart for kerf width.

The results of the ANOVA for kerf width are shown in Table 5. FPP and LCS are the most significant process parameters affecting kerf width, while LOP has the least effect. The results of the ANOVA for kerf width are also shown graphically in Figure 9, the Pareto chart. The observed value for the coefficient of determination (R^2^) indicates a high degree of fit between the model and the experimental data.

**Figure 10:**
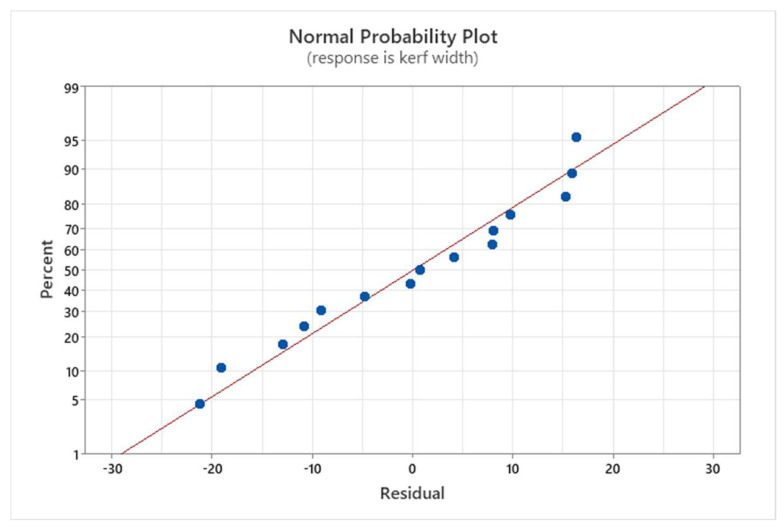
Normal Probability Plot for the kerf width.

Figure 10 presents the Normal Probability Plot for the kerf width. Observing that the data points align approximately linearly along the regression line, we can conclude that the data follows a normal distribution and that the linear regression model fits the experimental data well. The coefficient of determination (R^2^) is 97.28% for kerf width which shows the good quality of the interpolated equation.

According to Equation (2), the regression equation for the kerf width of the Hardox 400 sheet is as follows:

Kerf width = 604.6 + 0.0135 LOP − 0.0652 LCS + 28.0 FPP + 33.17 FPP × FPP(2)

**Figure 11:**
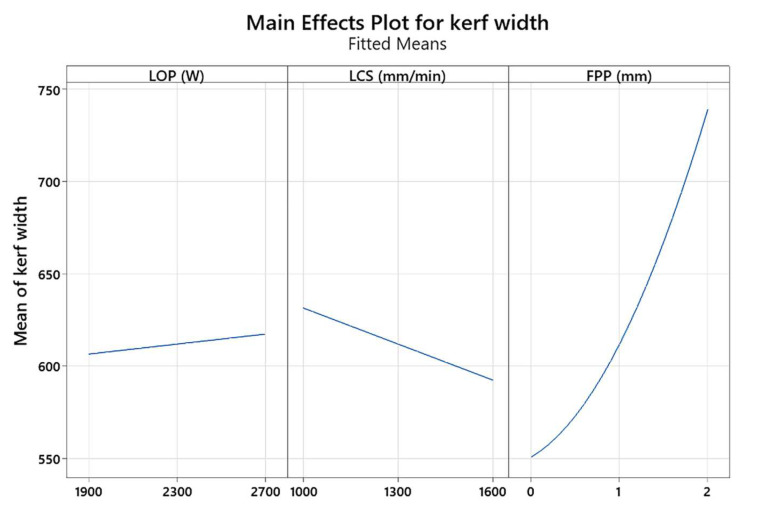
Effect of main parameters on kerf width.

Figure 11 shows the effect of the main parameters such as LOP, LCS, and FPP on the kerf width. It is seen that the kerf width is increased after increasing the LOP. The reason for this is that by increasing the LOP, the amount of thermal energy entered into the cut area increases, and then the volume of the molten area increases, which leads to an increase in the kerf width. Also, with increasing the positive FPP, the kerf increases. This is because the laser beam is focused above the workpiece, allowing the oxygen gas to react exothermically with the workpiece more easily, adding another heat energy source to the cutting process. With the increase in the amount of energy beam and the creation of a larger molten zone, the kerf increases. With increasing the LCS, the kerf decreases. This is because the high-energy laser beam passes through the cutting zone more quickly and the heat energy does not stay, so less material is melted in the cutting zone.

**Figure 12:**
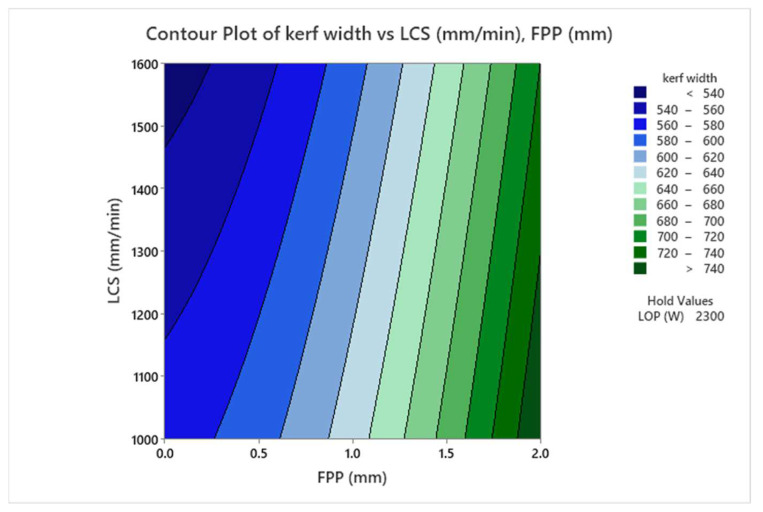
Contour plot of the interaction effects of FPP and LCS on kerf width.

Figure 12 presents the contour plot of the interaction effects of LCS and FPP on kerf width. The interaction plot indicates that kerf width decreases with increasing the LCS and also with decreasing the positive FPP towards the sheet surface. Increasing the LCS reduces the concentration of the heat energy impact point, and the FPP approaching the cutting surface prevents the oxygen gas from having optimal exothermic reaction conditions with the workpiece, reducing the heat energy and consequently resulting in less melting in the kerf.

### 3.3. Process Optimization

**Figure 13:**
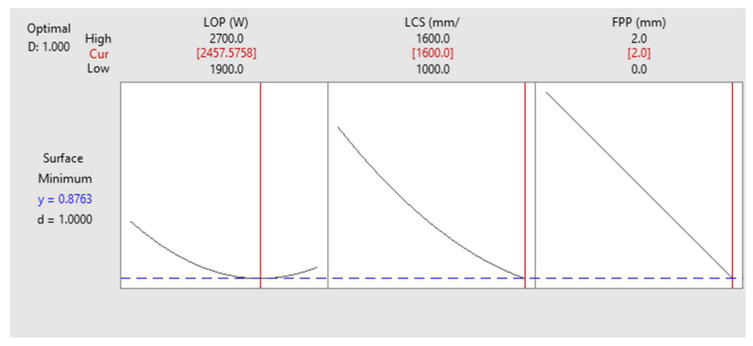
Surface Roughness Optimization of the cutting area.

The effect of process parameters on the characteristics of the cutting area in the LCP is very complex. This complexity arises from the fact that changes in various parameters in LCP affect both surface roughness and kerf width. Figure 13 shows the optimization settings for the minimum surface roughness. The results show that if only surface roughness optimization is considered, the optimal average surface roughness is around 1 µm if the LOP is 2457 watts, the LCS is 1600 mm/min, and the FPP is +2 mm above the sheet surface.

**Figure 14:**
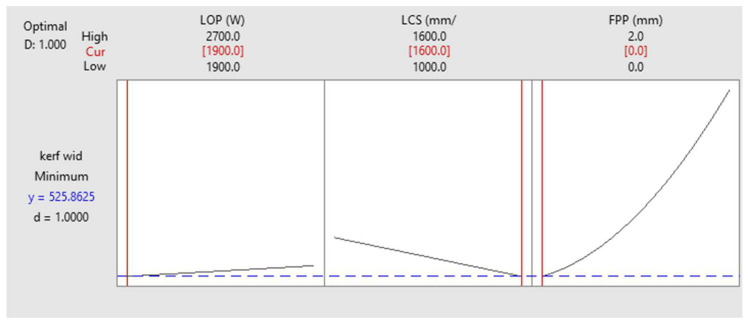
Kerf Optimization of the cutting area.

Figure 14 shows the optimization settings for the minimum kerf. The results show that if only kerf optimization is considered, the optimal kerf is 0.525 mm if the LOP is 1900 W, the LCS is 1600 mm/min, and the FPP is zero.

**Figure 15:**
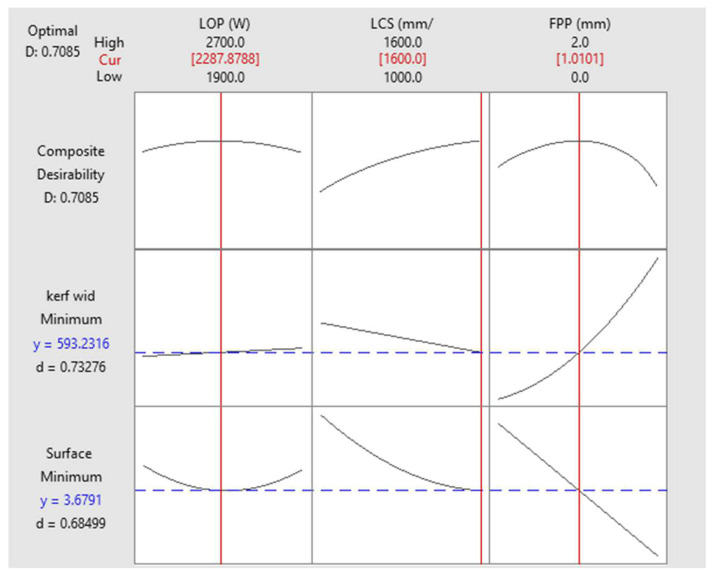
Optimization of both surface roughness and kerf width.

In the optimization of process parameters, one of the best choices for optimization is to minimize both surface roughness and kerf width together. The results of multi-objective optimization of the LCS of the Hardox 400 steel sheet in Figure 15 show that if the LOP is set to 2287 watts, the LCS is 1600 mm/min and the FPP is +1 mm above the sheet surface, the optimized surface roughness of the cut area will be 3.67 µm and the kerf width will be 0.593 mm.

Multi-objective optimization in manufacturing processes needs to consider the desirability function. As can be seen in Figure 13 and Figure 14, the optimum condition for individually minimizing the surface roughness and kerf width is different. This means that both of the output variables cannot be absolutely optimized and cannot be absolutely minimized concurrently. For a better understanding of the concept of multi-objective optimization, we need to define individual desirability function (*d*) and composite desirability function (*D*). Three types of optimization can be defined known as follows: smaller is the best (minimize), larger is the best (maximize), and nominal value is the best (target value). Individual desirability function (*d*) is defined as a scale in the range of [0, 1] according to the difference from maximum or minimum value. This method is proposed by Derringer and Suich and is applied in many pieces of optimization software such as Minitab. The individual desirability function (*d*) for the minimized condition is defined as Equation (3).

(3) $$d(y)={\left(\frac{y-U}{L-U}\right)}^{r}$$

where *r* is a user defined parameter (r>0). A zero value of *d* shows completely undesirability for the response and total desirability obtained when *d* is 1. Figure 13 and Figure 14 shows that the individual desirability is 1. But for combined optimization it is needed to define and use composite desirability function (*D*). The composite desirability function is calculated by the product (geometric mean) of the individual desirability function (*d*) of each output variable (Equation (4)).

(4) $$D(y)={\left(\prod_{j=1}^{n}{\left(d_{j}(y)\right)}^{w_{j}}\right)}^{\frac{1}{\sum_{j=1}^{n}w_{j}}}$$

where $d_{j}(y)$ is the individual desirability of *j*’th output variable and $w_{j}$ is the weight function of it. As can be seen in Figure 15, the individual desirability of surface roughness and kerf width is 0.68499 and 0.73276 and the composite desirability is 0.7085. This value of *d* and *D* shows that simultaneous optimization is just fair and better surface roughness or better kerf width can be obtained according to the desire of the industry. The readers can study [28,29] for more details and discussion about the desirability function in multi-objective optimization.

At last, it is worth noting that, the current study aims to find a relation between the process parameters (LOP, LCS, and FPP) and surface roughness and kerf width. This aim can be achieved by using deep learning approaches such as Support Vector Regression (SVR) methods, and meta-heuristic optimization methods (ANN, GA, PSO, …). These approaches need a high volume of data and the accuracy extremely depends on the volume of data. For example, 70% of the data will be used for the training of the neural network, and the remaining 30% used for evaluation of the trained network, while it cannot yield any closed-form equation for easy use. The DOE methods use a moderate volume of data and are appropriate for process optimization. Implementation of the new methods for prediction of the output process with more complicated models can be an attractive object for researchers.

## 4. Conclusions

In this experimental study, the laser-cutting process (LCP) of Hardox 400 steel sheets was investigated using the RSM and the regression model proposed a very high accuracy equation for prediction of surface roughness and kerf width. Input parameters of the LCP were selected as laser output power (LOP), laser-cutting speed (LCS), and focal point position (FPP), while surface roughness and kerf width were considered as output variables. The following results can be obtained from the present study:

- The results of the ANOVA for surface roughness and kerf width showed that the FPP and LCS are the most significant process parameters affecting the surface roughness and kerf width. After increasing the FPP, the surface roughness decreased and the kerf width increased. After increasing the LCS, the surface roughness and kerf width decreased.
- Regression equations for surface roughness and kerf width were obtained, which can significantly contribute to improving the LCP, increasing cut quality, reducing waste, increasing efficiency, and reducing costs.
- The normal probability plots and the coefficient of determination (R2) value for surface roughness and kerf width show that the proposed model by RSM can fit well with the experimental data.
- The results show that if only surface roughness is optimized, the optimal average surface roughness is around 1 µm obtained by 2457 W LOP, 1600 mm/min LCS, and +2 mm FPP conditions.
- The results show that if only kerf width is optimized, the optimal kerf width is 0.525 mm and obtained by 1900 W LOP, 1600 mm/min LCS, and zero FPP conditions.
- The multi-objective optimization to minimize both surface roughness and kerf width simultaneously was carried out and the results show that the optimized condition (2287 W LOP, 1600 mm/min LCS, and +1 mm FPP) leads to 3.67 µm surface roughness and 0.593 mm kerf width.

## Figures and Tables

**Table 1:** Chemical composition of Hardox 400 steel [5].

Alloy Element	Fe	C	Si	Mn	P	S	Cr	Ni	Mo	B
Weight Percent (%)	Base	0.32	0.70	1.60	0.025	0.010	2.50	1.50	0.60	0.004

**Table 2:** The levels of process parameters in LCP of Hardox 400 steel.

Input Variable	Units	Levels		
		I	II	III
LOP	W	1900	2300	2700
LCS	mm/min	1000	1300	1600
FPP	mm	0	+1	+2

**Table 3:** List of experiments for LCP of Hardox 400 according to RSM.

Experiment Number	LOP (W)	LCS (mm/min)	FPP (mm)
1	2300	1000	2
2	1900	1000	1
3	2300	1300	1
4	2300	1300	1
5	2300	1000	0
6	1900	1300	2
7	2700	1600	1
8	2700	1300	2
9	2300	1300	1
10	2300	1600	0
11	1900	1300	0
12	2700	1300	0
13	1900	1600	1
14	2700	1000	1
15	2300	1600	2

**Table 4:** ANOVA results for surface roughness.

Source	DF	Adj SS	Adj MS	F-Value	*p*-Value
Model	7	54.0436	7.7205	28.06	0.000
Linear	3	44.6275	14.8758	54.07	0.000
LOP (W)	1	0.0450	0.0450	0.16	0.698
LCS (mm/min)	1	18.3012	18.3012	66.52	0.000
FPP (mm)	1	26.2812	26.2812	95.53	0.000
Square	2	4.6636	2.3318	8.48	0.013
LOP (W) × LOP (W)	1	3.3116	3.3116	12.04	0.010
LCS (mm/min) × LCS (mm/min)	1	1.6635	1.6635	6.05	0.044
2-Way Interaction	2	4.7525	2.3762	8.64	0.013
LOP (W) × FPP (mm)	1	1.6900	1.6900	6.14	0.042
LCS (mm/min) × FPP (mm)	1	3.0625	3.0625	11.13	0.012
Error	7	1.9258	0.2751		
Lack-of-Fit	5	1.7391	0.3478	3.73	0.225
Pure Error	2	0.1867	0.0933		
Total	14	55.9693			
S = 0.524509 R-sq = 96.56% R-sq (adj) = 93.12%					

**Table 5:** ANOVA results for kerf width.

Source	DF	Adj SS	Adj MS	F-Value	*p*-Value
Model	4	78,604.7	19,651.2	89.31	0.000
Linear	3	74,496.3	24,832.1	112.85	0.000
LOP (W)	1	234.4	234.4	1.07	0.326
LCS (mm/min)	1	3065.4	3065.4	13.93	0.004
FPP (mm)	1	71,196.5	71,196.5	323.56	0.000
Square	1	4108.4	4108.4	18.67	0.002
FPP (mm) × FPP (mm)	1	4108.4	4108.4	18.67	0.002
Error	10	2200.4	220.0		
Lack-of-Fit	8	2135.6	266.9	8.24	0.113
Pure Error	2	64.8	32.4		
Total	14	80,805.1			
S = 14.8337 R-sq = 97.28% R-sq (adj) = 96.19%					

## Data Availability

The original contributions presented in the study are included in the article, further inquiries can be directed to the corresponding author.
